# Data file of a deep proteome analysis of the prefrontal cortex in aged mice with progranulin deficiency or neuronal overexpression of progranulin

**DOI:** 10.1016/j.dib.2016.11.030

**Published:** 2016-11-19

**Authors:** Juliana Heidler, Stefanie Hardt, Ilka Wittig, Irmgard Tegeder

**Affiliations:** a Institute of Clinical Pharmacology, Goethe-University Hospital, Frankfurt, Germany; b Functional Proteomics, SFB815 Core Unit, Goethe-University, Frankfurt, Germany

**Keywords:** Proteomics, Progranulin, Cortex, Neurodegeneration, Aging

## Abstract

Progranulin deficiency is associated with neurodegeneration in humans and in mice. The mechanisms likely involve progranulin-promoted removal of protein waste via autophagy. We performed a deep proteomic screen of the pre-frontal cortex in aged (13–15 months) female progranulin-deficient mice (GRN^−/−^) and mice with inducible neuron-specific overexpression of progranulin (SLICK-GRN-OE) versus the respective control mice. Proteins were extracted and analyzed per liquid chromatography/mass spectrometry (LC/MS) on a Thermo Scientific™ Q Exactive Plus equipped with an ultra-high performance liquid chromatography unit and a Nanospray Flex Ion-Source. Full Scan MS-data were acquired using Xcalibur and raw files were analyzed using the proteomics software Max Quant. The mouse reference proteome set from uniprot (June 2015) was used to identify peptides and proteins. The DiB data file is a reduced MaxQuant output and includes peptide and protein identification, accession numbers, protein and gene names, sequence coverage and label free quantification (LFQ) values of each sample. Differences in protein expression in genotypes are presented in "Progranulin overexpression in sensory neurons attenuates neuropathic pain in mice: Role of autophagy" (C. Altmann, S. Hardt, C. Fischer, J. Heidler, H.Y. Lim, A. Haussler, B. Albuquerque, B. Zimmer, C. Moser, C. Behrends, F. Koentgen, I. Wittig, M.H. Schmidt, A.M. Clement, T. Deller, I. Tegeder, 2016) [1].

**Specifications Table**

**Table:** 

Subject area	*Neuroscience, Proteomics*
More specific subject area	*Neurobiology, Aging, Frontal cortex*
Type of data	*Tables*
How data was acquired	*Liquid chromatography/mass spectroscopy.* Thermo Scientific™ Q Exactive Plus equipped with an ultra-high performance liquid chromatography unit and a Nanospray Flex Ion-Source
Data format	Table 1: Raw MaxQuant data
	Table 2: Gene Ontology analysis
Experimental factors	*Tissue dissection, freezing on dry ice and storage at* −80 °C*, protein extraction, chromatography and MS analysis, MaxQuant software and Perseus software, Gene Ontology annotation with DAVID*
Experimental features	*Aged female GRN-/- mice versus controls and tamoxifen inducible SLICK-GRN-OE mice with/without tamoxifen treatment. The last tamoxifen treatment 4 months before tissue dissection*
Data source location	*Frankfurt, Germany*
Data accessibility	*Data is with this article and raw MS data, a full MaxQuant table and a description of the sample processing are deposited to the ProteomeXchange Consortium*[2]*via the PRIDE partner repository with the dataset identifier PRIDE:*PXD004087*, which are publicly available.*
	*Project Webpage:*
	http://www.ebi.ac.uk/pride/archive/projects/PXD004087
	*FTP Download:*
	ftp://ftp.pride.ebi.ac.uk/pride/data/archive/2016/10/PXD004087

**Value of the data**

• The proteome data are useful to gain further insight into the functions of progranulin in the context of aging before manifestations of behavioral manifestations of neurodegeneration.
• The data are useful to build protein networks and reveal novel biological functions, in which progranulin is involved.
• The data may be used for comparison of proteome sets of the prefrontal cortex and other regions of the brain of mice and humans.

## 1 Data

The data is a reduced MaxQuant output file including peptide and protein identification, accession numbers, protein and gene names, sequence coverage and label free quantification (LFQ) values of each sample. Identifications from the reverse decoy database, identified by site only and known contaminants were excluded. The full table and the raw mass spectrometry results are available with the PRIDE Project PXD004087.

In addition, the Excel file contains a table with the Gene Ontology terms associated with the proteins, which were differentially expressed between groups using Volcano Plots (Perseus software, *P*<0.05 FDR adjusted). The GO overrepresentation analysis was done with DAVID.

## 2 Experimental design, materials and methods

We performed a deep proteome analysis of the prefrontal cortex in aged Grn^+/+^ (wild type) and Grn^−/−^ (progranulin knockout) mice and in mice with tamoxifen inducible Thy1 driven neuronal overexpression of progranulin, referred to as SLICK-GrnOE&TAM versus SLICK-Grn mice (treated with vehicle) as described in [1]. Three mice were used per group. The processing of the samples is described in detail with the PRIDE Project PXD004087.

Briefly, frozen tissue samples were homogenized in 20% trichloroacetic acid and proteins were solubilized in extraction buffer (10% SDS, 150 mM NaCl, 50 mM Tris–HCl pH 7.6) and sonified. Protein content was measured by Lowry´s assay. Proteins were digested using the Filter Aided Sample Preparation (FASP) [3] and peptides were acidified with TFA and fractionated by C18/SCX stage tips [4].

Liquid chromatography/mass spectrometry (LC/MS) was performed on Thermo Scientific™ Q Exactive Plus equipped with an ultra-high performance liquid chromatography unit (Thermo Scientific Dionex Ultimate 3000) and a Nanospray Flex Ion-Source (Thermo Scientific). Peptides were loaded on a C18 reversed-phase pre-column (Thermo Scientific) followed by separation with 2.4 µm Reprosil C18 resin (Dr. Maisch GmbH, Germany) in-house packed into picofrit emitter tips (New Objectives) using a gradient from 5% mobile phase A (4% acetonitrile, 0.1% formic acid) to 30% mobile phase B (80% acetonitrile, 0.1% formic acid) for 110 min followed by a second gradient until 60% B for 45 min with a flow rate 300 nl/min, and subsequent washout. MS data were recorded by selecting the most abundant precursor ions in positive mode for HCD fragmentation. The full MS scan range was 300–2000 m/z with resolution of 70,000, and an automatic gain control and a maximal ion injection time of 160 ms. Only higher charged ions (2+) were selected for MS/MS scans with an isolation window of 2 m/z. Full Scan MS-data were acquired in profile mode using Xcalibur software.

Xcalibur raw files were analyzed using the proteomics software Max Quant (1.5.2.8) [5]. The mouse reference proteome set from uniprot (Download June 2015, 76086 entries) was used to identify peptides and proteins. The false discovery rate (FDR) was set to 1%. The data file includes peptide and protein identification, accession numbers, protein and gene names, sequence coverage and label free quantification (LFQ) values of each sample. Identifications from the reverse decoy database, identified by site only and known contaminants were excluded.

Proteomic data were analyzed with Perseus 1.5.2.6 [6] as described in [1]. The control groups (Grn^+/+^ and SLICK-Grn without tamoxifen) were similar and were combined to gain power. Ontology annotations of significantly regulated proteins for “cellular component”, “biological process” and “molecular function” were analyzed to assess common localizations and functions as described in [1].

## Footnotes

Appendix A Supplementary data associated with this article can be found in the online version at http://dx.doi.org/10.1016/j.dib.2016.11.030.
