# Supplementary material for: Data file of a deep proteome analysis of the prefrontal cortex in aged mice with progranulin deficiency or neuronal overexpression of progranulin
Source: Data Brief. 2016 Nov 19;9:1070–3. doi: 10.1016/j.dib.2016.11.030 (PMC5126127; doi:10.1016/j.dib.2016.11.030)
Supplement: Supplementary file 1 — Supplementary material [file mmc1.pdf]

Conflicts of Interest Statement

Manuscript title: Data file of a deep proteome analysis of the prefrontal cortex in aged mice with progranulin deficiency or neuronal overexpression of progranulin

The authors whose names are listed immediately below certify that they have NO affiliations with or involvement in any organization or entity with any financial interest (such as honoraria; educational grants; participation in speakers' bureaus; membership, employment, consultancies, stock ownership, or other equity interest; and expert testimony or patent-licensing arrangements), or non-financial interest (such as personal or professional relationships, affiliations, knowledge or beliefs) in the subject matter or materials discussed in this manuscript.

Author names:

Irmgard Tegeder: Corresponding author signing on behalf of all authors

Other authors: Juliana Heidler, Stefanie Hardt, Ilka Wittig
email addresses:
Irmgard Tegeder: itegeder@hotmail.com
Juliana Heidler: julianaheidler@googlemail.com
Stefanie Hardt: stefanie.hardt@live.de
Ilka Wittig: wittig@med.uni-frankfurt.de
